# Improving Steam Methane Reforming Efficiency via Hierarchical Structure in Additively Manufactured Ni-Based Self-Catalytic Reactors

**DOI:** 10.3390/ma18061350

**Published:** 2025-03-19

**Authors:** Dongdong Dong, Jiangqi Zhu, Min Liu, Xingchen Yan, Bingwen Lu, Kesong Zhou

**Affiliations:** 1 School of Materials and Energy, Guangdong University of Technology, Guangzhou 510006, China; dongdd1990@163.com; 2 Guangdong-Hong Kong Joint Laboratory of Modern Surface Engineering Technology, Guangdong Provincial Key Laboratory of Modern Surface Engineering Technology, Institute of New Materials, Guangdong Academy of Sciences, Guangzhou 510651, China

**Keywords:** self-catalytic reactor, additive manufacturing, steam reforming of methane

## Abstract

Hydrogen is an ideal feedstock fuel for solid oxide fuel cells (SOFCs). The steam reforming of methane (SRM) is the predominant method of producing hydrogen. However, the process of SRM relies on the involvement of a catalyst, and the reforming efficiency is constrained by the limited surface area in the traditional catalyst system. In this study, a mixer structure is applied to improve the mixing of the methane. Nano-sized pores are introduced to the struts of the mixer structure, forming a hierarchical structure, to effectively reduce the weight and increase the surface area of the self-catalytic reactors, hence increasing the catalytic efficiency. The hierarchical structure increases the reforming efficiency at all temperatures, and the level of improvement reaches its peak when the conversion rate of methane increases by 192% at 800 °C and by 40% at 900 °C compared to the self-catalyst without a hierarchical structure.

## 1. Introduction

A solid oxide fuel cell (SOFC) can generate electric or thermal power with an extremely high conversion efficiency (70% [1] even 80% [2]) and minimize the emission of a pollution side product. Due to the pursuit of clean energy, green fuels with high efficiency (e.g., hydrogen) show great potential as an energy source for SOFCs [3]. Hydrogen is one of the most fascinating energy sources being pursued due to its higher calorific value compared to conventional fossil fuels and clean combustion products. To meet the growing demands of hydrogen, the stable and efficient production of hydrogen is critical. The reforming of methane is a common and desired method to convert the greenhouse gas to high value-added H_2_ or syngas [4]. Therefore, among all the methane-to-hydrogen conversion strategies, the steam reforming of methane (SRM) predominates in the hydrogen industry [5]. Hydrogen is fabricated by an endothermic reaction between water steam and methane at temperatures ranging from 750 °C to 950 °C and under the pressure of 14–20 bar (Equations (1) and (2)) [6,7].

(1) $$CH_{4}+H_{2}O⇌CO+3H_{2}$$

(2) $$H_{2}O+CO⇌{CO}_{2}+H_{2}$$

Catalysts are indispensable in the practical fabrication of methane via SRM as they substantially increase the efficiency of the reaction. Catalysts, such as Co/Al_2_O_3_ [8] and Rh/Al_2_O_3_ [9], are composed of metallic particles as the catalyst and γ-alumina as the carrier (also called the reactor or supporter) of the metallic particles. To date, the Ni/Al_2_O_3_ catalyst is the most widely employed in SRM due to its high efficiency and low cost [5]. The efficiency of the catalyst is dependent on the specific surface area, which provides the reaction sites. Hence, to increase the specific surface area, traditional catalysts are essentially Ni particles distributed on the porous γ-alumina reactor. Such a porous structure requires multiple manufacturing processes including the fabrication of the catalyst, the reactor, and the integration process. The complex fabrication process and the homogeneous distribution of the catalyst particles remain challenging. In addition, the structure of the traditional reactor only acts as a supporter for the catalyst, and its design does not fully consider the surface area and the mixing of the reactants; hence, the catalytic effect of the catalyst is not fully activated [10].

Manipulating the flow of reactants is the key to improving the mixing process. Inspired by the excellent mixing effect induced by a static mixer, the SX-type structure, one of the most effective structures of the mixer, was incorporated into the self-catalytic system [11]. The SX-type structure is composed of struts intersecting with each other, introducing turbulence to the liquid or gas passing by. Thereby, the SX structure shows a great mixing effect on the laminar flow [12]. Based on the features of the mixing structure, the integration of the mixer structure with the reactor shows the potential to improve the mixing of the reactants. However, fabricating the SX-type structure is challenging and costly using conventional processing methods. Therefore, a more advanced method for the functional integration of the catalyst and reactors is needed.

Additive manufacturing (AM) is a novel processing technology that enables the fabrication of components with complex lattice structures. With the aid of AM technology, reactors with a complicated lattice structure can be readily fabricated in one AM process. In addition, the catalyst, Ni, is integrated into the reactor, namely a self-catalytic reactor, by AM technology at the same time [10]. By fabricating the catalyst and reactor into a whole component, the integration issue of the catalyst and reactor could be solved. To date, the application of additive manufacturing for methane reforming is still nascent. Most of the research focuses on the fabrication of porous reactors for various reactions [13,14]. However, the reaction efficiency of the additively manufactured (AMed) reactor is not better than that of the traditional reactor, which is mainly attributable to the fact that the reaction area is still not inadequate and the advantages of AM on the structure design have not been fully implemented [13,14]. Therefore, improving the specific surface area of the reactor is critical to enhancing the performance of the AMed catalyst system.

In this study, nickel-based self-catalytic reactors composed of an SX structure are fabricated via AM technology. To increase the reaction area, the micron-sized pores are introduced to the struts of the SX structure, forming a hierarchical structure. Such a structure is expected to force the sufficient mixing of the reactants and increase the reaction area. However, introducing nano-sized pores is beyond the processing accuracy of AM technology due to the limitation of the laser spot size.

Balling is an unexpected phenomenon that occurs during the process of AM due to insufficient energy input to induce the completed spreading of the melt. The metallic melt that is not completely spread tends to curl up and thus breaks the linkages between neighboring fabricated parts. As a result, the incomplete spreading of the melt introduces a massive amount of lack-of-fusion (LOF) defects in the consequent component, leading to a decrease in the mechanical properties [15]. In this study, the balling behavior is carefully manipulated by processing optimization and hence constrains the size of LOF defects. By taking advantage of this method, micron pores are introduced into the struts of the lattice structure. By evaluating and analyzing the methane reforming efficiency, the performance and the underlying mechanism of the SRM reaction by the AMed self-catalytic reactor with a hierarchical structure is revealed, facilitating the future development of AM technology in traditional chemical industries.

## 2. Experimental Methods

### 2.1. Materials

**Figure 1:**
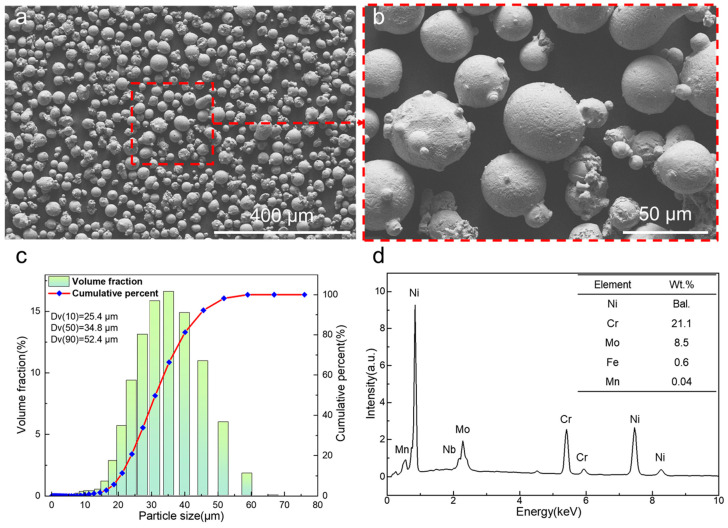
(**a**,**b**) Micrograph, (**c**) size distribution and (**d**) EDX point spectra of Inconel 625 powder.

Laser powder bed fusion (LPBF) was used to fabricate the Ni-based self-catalytic reactors. Gas-atomized Inconel 625 powder (Carpenter^®^, Reading, PA, USA) was used as feedstock. The powder morphology shows a near-spherical shape with minor satellite powder attached to the powder surface (Figure 1a,b). The size distribution and elemental compositions are shown in Figure 1c,d. The particles show a concentrated distribution in size with a Dv(10) of 25.4 μm, Dv(50) of 34.8 μm and Dv(90) of 52.4 μm. Apart from Ni, the IN625 powder contains 21.1% of Cr, 8.5% of Mo, 0.6% of Fe, and 0.04% of Mn (all in wt.%).

### 2.2. Structure Design

**Figure 2:**
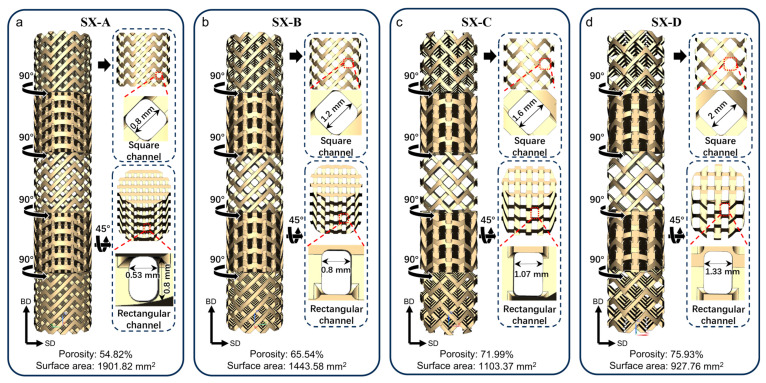
A schematic of the self-catalytic reactor constructed by (**a**) SX-A, (**b**) SX-B, (**c**) SX-C and (**d**) SX-d structures.

Four types of SX-type lattice structures denoted as SX-A, SX-B, SX-C and SX-D are studied in this work, as shown in Figure 2. The whole self-catalytic reactor has a cylinder shape composed of 5 units with the SX-A structure connecting end to end with an interval rotation of 90°. Each unit has a diameter of 10 mm and a length of 40 mm. The unit with the SX structure contains square and rectangular channels for the reactants to flow and mix. The size of the channel is the only difference among all units, and the geometric dimension of the channel is demonstrated in Figure 2. As the cross-sectional size of all struts is maintained identically as 0.6 mm × 0.6 mm, the number of channels increases as the size of the channels increases. The size of the square channels increases from 0.8 mm to 2 mm with an interval of 0.2 mm, and the width of the rectangular channel increases from 0.53 mm to 1.33 mm from SX-A to SX-D, while the length of the rectangular channel remains the same for all SX structures. The surface area and porosity of the SX-A to SX-D structures were calculated from the digital model, and the calculated surface area decreased and the porosity (i.e., the volume fraction of the channels) increased from SX-A to SX-D.

### 2.3. Fabrication Process

**Figure 3:**
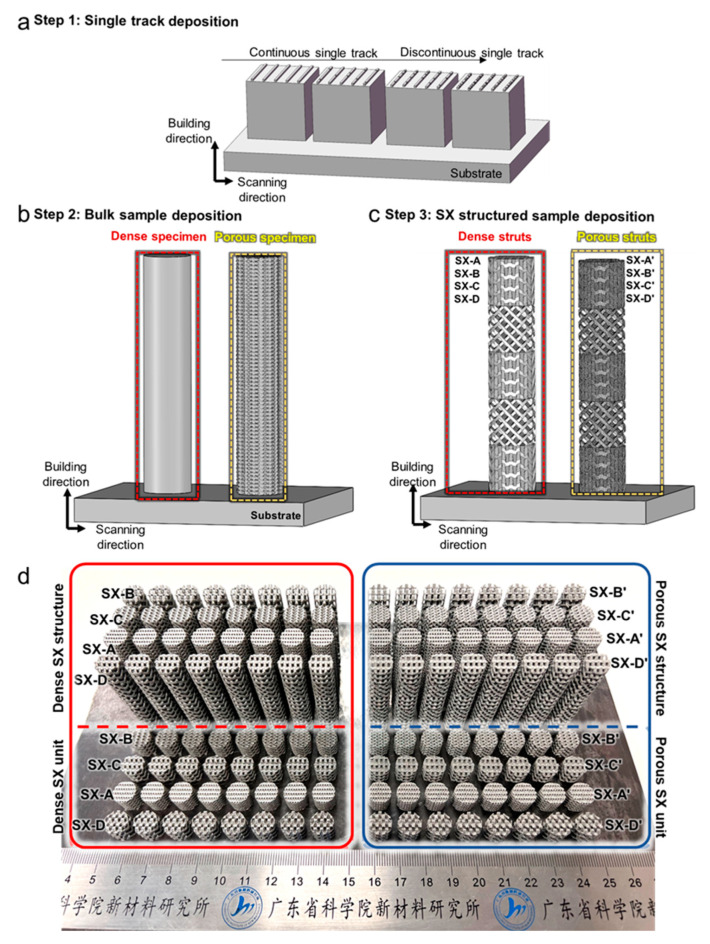
A schematic of the processing optimization: (**a**) single-track deposition, (**b**) bulk sample deposition and (**c**) SX-structured sample deposition. (**d**) Photographs of porous and dense SX-structured specimens and compression specimens.

The samples were fabricated using laser powder bed fusion (LPBF) technology with the EOS M290 (EOS, Krailling, Germany). The fabrication process of the self-catalytic reactor is illustrated in Figure 3. The processing parameters used to fabricate SX structures with dense struts are adopted from a previous study [16]. For the processing parameters used to fabricate the SX structure with porous struts, the processing optimization was conducted following the procedure depicted in Figure 3. The cubic substrates were fabricated by LPBF. The laser power and scanning speed for the fabrication of porous lattice struts were determined by single-track processing (Figure 3a). Subsequently, the processing parameters were selected to fabricate the bulk specimen, examining their formability (Figure 3b). Finally, the self-catalytic specimens with SX structures were fabricated using the optimum processing parameters. For the self-catalytic specimens, the lattice structures were fabricated with porous struts by selecting different processing parameters. The specimens that have dense struts are denoted as SX-A, SX-B, SX-C and SX-D. Similarly, the specimens that have porous struts (namely a hierarchical structure) are denoted as SX-A’, SX-B’, SX-C’ and SX-D’ (Figure 3d). All the processing parameters used in this study are listed in Table 1.

### 2.4. Microstructure Characterization and Mechanical Property Tests

Cubic specimens with a dimension of 10 mm × 10 mm × 10 mm were fabricated for microstructure characterization. All specimens were extracted from the substrate using electric discharge machining (EDM). The specimens were ground using 600, 800, 1200, 2500, and 4000 grit sandpapers and then polished using the active oxide polishing suspension (OPS) colloidal silica (0.05 μm) to remove the deformed surface layer.

The phase identification was conducted using Bruker D8 Advance X-ray diffraction (Guangdong Academy of Science, Guangzhou, China), with the diffraction angles (2θ) ranging from 20° to 110°, and the specimens were scanned at a scan speed of 3°/min using Cu Kα radiation at 40 kV and 40 mA. The form of the elements before and after the reforming reaction was identified using X-ray photoelectron spectroscopy (XPS, EscaLab Xi+, West Sussex, UK). The XPS tests were conducted using a standard Al target X-ray source (1486.6 eV) in a vacuum of 5 × 10^−9^ mbar, and C 1s was used as a reference for the energy calibration.

The morphology of the melt pool was characterized using optical microscopy (Leica DM750, Wetzlar, Germany). The microstructure of the polished sample and the surface morphology of the AMed lattice struts were characterized using a scanning electron microscope (FEI-Nova NanoSEM 430, Hillsboro, OR, USA) equipped with an energy-dispersive X-ray (EDX) detector.

The compressive properties of the SX structure unit were tested. The dimensions of the SX structure unit for the compression test are 10 mm in diameter and 10 mm in length, as shown in Figure 3d. The compression rate remained constant at 0.375 mm/min, and every structure was measured 3 times.

### 2.5. Catalyst Property Tests

**Figure 4:**
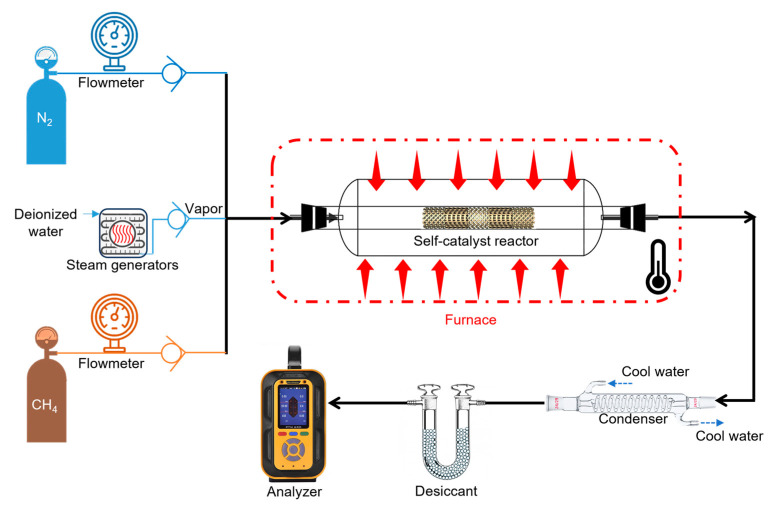
A schematic of the catalyst system for the catalyst property test.

The surface area of the specimens with a hierarchical structure (i.e., SX-A’ to SX-D’) was measured using Autosorb Station 1 (Quantachrome^®^, Boynton Beach, FL, USA) via the physical adsorption of nitrogen based on the Brunauer–Emmett–Teller (BET) theory. The catalyst properties are tested in a sealed glass tube, with the reactant gas and protective gas pumped into the glass tube from the inlet and the reaction product flowing out from the outlet as shown in Figure 4. CH_4_ and N_2_ were pumped into the water vaporizer, in which the deionized water is vaporized. Then, the reactants (CH_4_ and H_2_O) were injected into the glass tube, mixing with N_2_. N_2_ was not involved in this reaction, and thus, the volume of N_2_ was considered to be unchanged throughout the reaction; therefore, it acted as an indicator for the reaction efficiency of the SRM reaction. The outlet gas was cooled down and dried before the compositional test using a gas analyzer. The reforming reaction was conducted subsequently in a tube furnace at 500 °C, 600 °C, 700 °C, 800 °C, and 900 °C. When the temperature reached the target temperature and the flow rate of the outlet gas stabilized without an apparent fluctuation, the self-catalytic reactor was preserved for 30 min and the data were collected every 10 min for the measurement of catalytic property.

The flow rates of CH_4_, H_2_O and N_2_ are 50 mL/min, 0.081 mL/min and 10 mL/min, respectively, as rationalized in the Supplementary Materials. The flow rates of CH_4_ and N_2_ are measured using flowmeters, and the flow rate of the deionized water is controlled by a syringe pump (Infusetek^®^, Shanghai, China). The flow rate of N_2_ at the outlet ($Q_{N_{2}-out}$) is supposed to be 10 mL/min. Based on the volume fraction of N_2_ ($V_{\%-N_{2}}$), the flow rate of one of the products X ($Q_{X-out}$) is calculated by the following equation:

(3) $$Q_{X-out}=Q_{N_{2}-out}\times V_{\%-X}/V_{\%-N_{2}}$$

The conversion rate of methane ($\alpha_{{CH}_{4}}$) is defined as the ratio of CH_4_ involved in the reforming reaction. The outlet CH_4_ represents CH_4_ that does not participate into the reaction. Therefore, the ratio of CH_4_ involved in the reforming reaction ($\alpha_{{CH}_{4}}$) is calculated as follows:

(4) $$\alpha_{{CH}_{4}}=\left(1-Q_{{CH}_{4}-out}/Q_{{CH}_{4}-in}\right)\times 100\%$$

The selectivity of CO ($S_{CO}$) is an indicator used to reflect the extent of the reaction of the major reaction (Equation (1)), which is represented by the amount of CH_4_ that is converted into CO. It is calculated based on the ratio of outlet CO over the reacted CH_4_, as shown in the equation below:

(5) $$S_{CO}=Q_{CO-out}/(Q_{{CH}_{4}-in}-Q_{{CH}_{4}-out})$$

Similarly, the selectivity of H_2_ ($S_{H_{2}}$) is calculated as follows:

(6) $$S_{H_{2}}=Q_{H_{2}-out}/\left\{2\left(Q_{{CH}_{4}-in}-Q_{{CH}_{4}-out}\right)+Q_{CO-out}+Q_{{CO}_{2}-out}\right\}$$

And the yield rate of H_2_ ($Y_{H_{2}}$) is calculated as

(7) $$Y_{H_{2}}=S_{H_{2}}\times \alpha_{{CH}_{4}}\times 100\%$$

## 3. Results and Discussions

### 3.1. Processing Optimization

**Figure 5:**
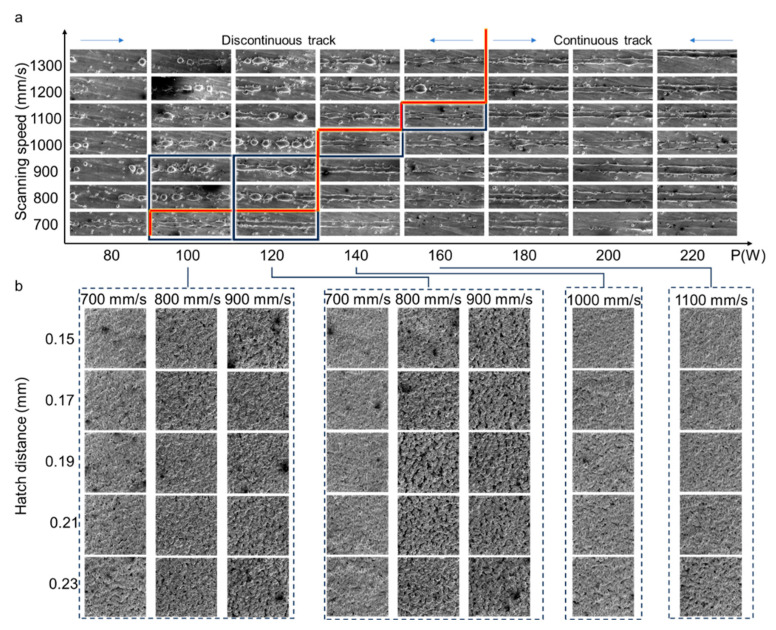
Micrographs of (**a**) continuity of single tracks fabricated using various processing parameters and (**b**) bulk specimens fabricated by processing parameters highlighted by blue outline in (**a**). The orange line represents the border of the continuous and discontinuous single track.

A large range of processing parameters is applied to the fabrication of a single track. When the power is low, the single track is discontinuous due to the balling effect, and the melt pool is not visible (Figure 5a). With an increase in laser power, the continuity of the single track improved. The increased laser power induces more spreading of the Ni melt, and the balling is eliminated. However, for a given power, an increase in the scanning speed also makes the melt pool become unstable. Therefore, to ensure the feasibility of fabricating the porous struts, the processing parameters in the proximity of the border of continuous and discontinuous tracks are selected to fabricate the bulk specimens with various hatch distances ranging from 0.15 to 0.23 mm, with an interval of 0.02 mm (Figure 5b).

For all processing parameters, increasing the hatch distance leads to more defects in the specimens. It is shown that the bulk sample is fabricated by processing parameters that deposit continuous tracks results in a relatively dense bulk specimen, such as the specimens fabricated by the power of 140 and 160 W and hatch distance of 0.15–0.19 mm. The defects are minor and mostly small in size with an inhomogeneous distribution (Figure 5b). The morphology of the surface indicates that the spreading of the melt pool leads to more bonding between the adjacent continuous tracks [16]. Therefore, the processing parameters with high power are not suitable for the fabricated porous struts. In contrast, processing parameters that fabricate discontinuous tracks result in more LOF defects. For the low hatch distance, the spreading of the melt pool is able to induce bonding between the adjacent tracks despite the balling, resulting in a similar morphology to those comprised by continuous tracks. With the increase in hatch distance, the LOF defects increase and form completed porous specimens, such as the specimens fabricated at 120 W and scanning speeds of 800 mm/s and 900 mm/s. Among all the bulk specimens, specimens fabricated at a power of 120 W, scanning speed of 800 mm/s and hatch distance of 0.21 mm exhibit a homogeneous distribution of pores, with concentrated sizes ranging from 50 to 250 μm. By further decreasing the power or increasing the scanning speed, the LOF defects tend to agglomerate, which might lead to the catastrophic failure of the whole structure. The enlarged micrographs show that the balling effect of the track due to the low power induces a large number of LOF defects, forming a porous specimen, as shown in the figures of bulk specimens fabricated at a power of 120 W and scanning speed of 900 mm/s. In addition, the surface of the discontinuous track is attached with nano-sized powder, which further increases the surface area for the reaction. Therefore, the processing parameters (a power of 120 W, scanning speed of 800 mm/s and hatch distance of 0.21 mm) are selected to fabricate the SX-structured specimens with porous struts.

### 3.2. Hierarchical Structure

**Figure 6:**
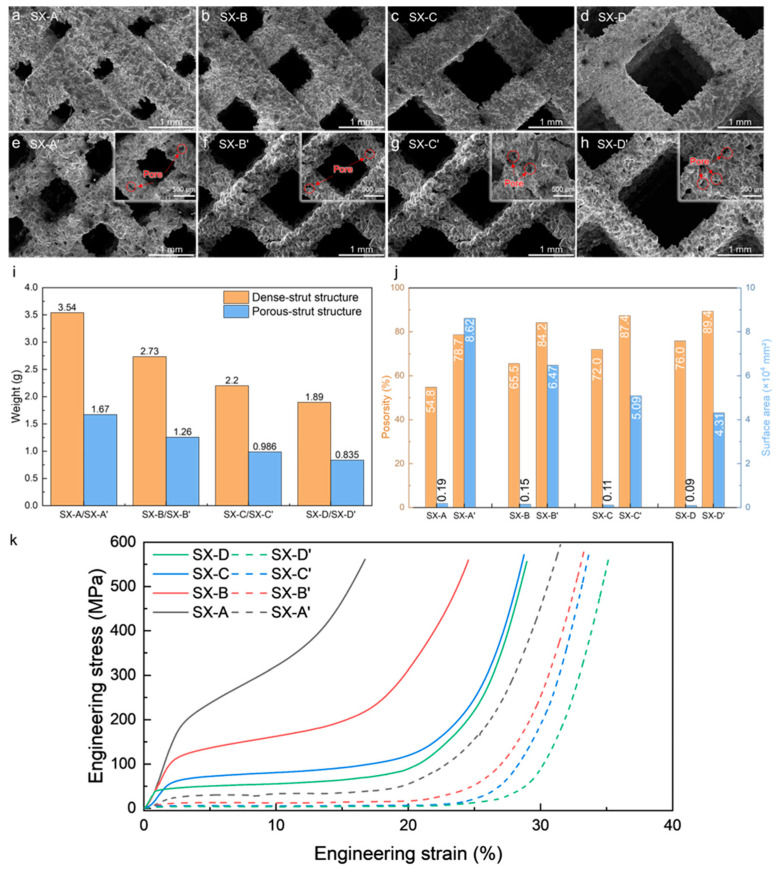
SEM micrographs of (**a**) SX-A, (**b**) SX-B, (**c**) SX-C, (**d**) SX-D, (**e**) SX-A’, (**f**) SX-B’, (**g**) SX-C’ and (**h**) SX-D’. The inset highlights the pores on the struts. The (**i**) weight, (**j**) porosity and surface area of the LPBFed SX structure specimen. (**k**) Tensile test results of SX-structured specimens.

The selected processing parameters show good formability and forming accuracy as the SX structure with dense and porous struts exhibits similar structures and geometric sizes. For the specimens with the same SX structure, the channels for the mixing of reactants are successfully fabricated (Figure 6a,e). The dense-strut specimens exhibit a fully dense microstructure with the size of the struts being slightly thicker due to more spreading of the melt pool (Figure 6a–d). For the porous-strut specimens, micron-sized pores with an average size of less than 100 μm induced by the LOF defects were introduced into the struts (Figure 6e–h), indicating the formation of a hierarchical structure. Due to the balling effect of the discontinuous single track, the struts of the hierarchical SX structure are slightly thinner than those of the dense-strut SX structure. The size of the channels of SX-A, SX-B, SX-C and SX-D is measured to be 0.35 mm, 0.75 mm, 1.20 mm, and 1.45 mm. As for the hierarchical structure, the sizes of the channels of the SX-A’, SX-B’, SX-C’ and SX-D’ specimens are 0.65 mm, 1.00 mm, 1.45 mm and 1.70 mm, respectively. It is noteworthy that the surfaces of the struts of the SX-A’, SX-B’, SX-C’ and SX-D’ specimens have a large amount of particles attached. Due to the low energy input, the powder is partially melted and attached to the struts. The attachment of the powder also increases the surface area for CH_4_ to reform.

Due to the formation of the pores in a hierarchical structure, the SX-A’, SX-B’, SX-C’ and SX-D’ specimens exhibit a significant decrease in mass compared to the dense-strut specimens. The mass of the SX-A, SX-B, SX-C and SX-D specimens is 3.5, 2.7, 2.1 and 1.8 g, receptively. The decrease in the mass is due to the increase in the size of the channels. The introduction of the hierarchical structure further reduces the weight by nearly 50%. The weight of the SX-A’, SX-B’, SX-C’ and SX-D’ specimens is 1.7, 1.2, 2.2 and 0.8 g, respectively (Figure 6i). The porosity of the SX structure, calculated from the model in Figure 2, is 54.8%, 65.5%, 72.0%, and 76.0%, respectively. Additionally, the porosity of the porous-strut specimens increases significantly compared to the dense-strut specimens. The porosity increased to 78.7%, 84.2%, 87.4%, and 89.4% for SX-A’, SX-B’, SX-C’ and SX-D’, respectively (Figure 6j). As a result of the hierarchical structure, the surface area, measured by the BET theory, of the SX-A’, SX-B’, SX-C’, and SX-D’ specimens increases drastically compared to that of the dense-strut specimens (Figure 6j). The increase in the surface area provides abundant reaction sites for the reforming reaction to initiate.

The mechanical properties of the SX-structured specimen with dense and porous struts are shown in Figure 6k. For all specimens, the structure with the largest channel size has the lowest strength but the largest elongation. Compared to the dense-strut specimens, namely SX-A, SX-B, SX-C and SX-D, the porous-strut specimens exhibit lower strength as the porous-strut SX structure deforms easier and exhibits lower strength. The increased porosity of the SX-A’, SX-B’, SX-C’ and SX-D’ specimens means that they have more space to collapse, and therefore, the elongation is increased. The decrease in the mechanical properties indicates the weakening effect of LOF defects in the hierarchical structure [17].

### 3.3. Catalyst Performance

**Figure 7:**
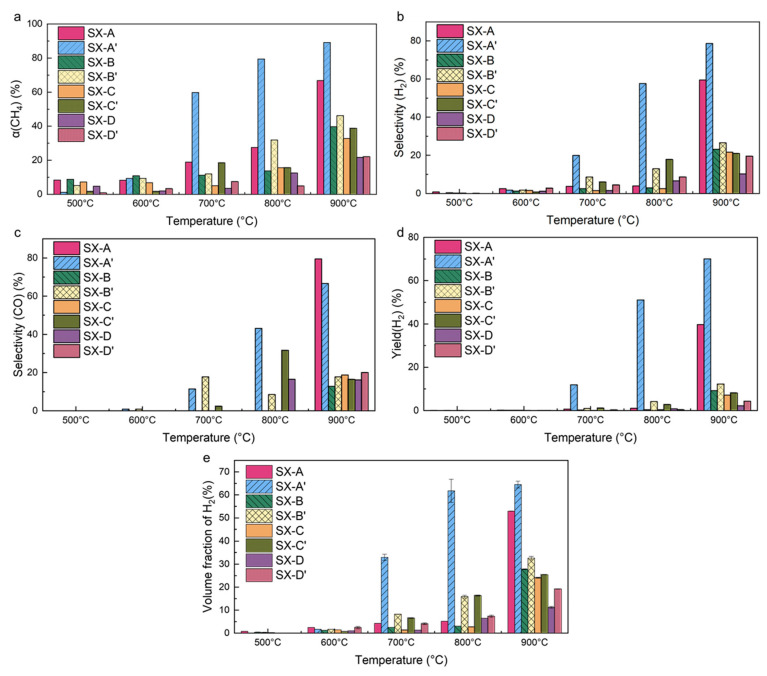
(**a**) Conversion rate of CH_4_, (**b**) selectivity of CO, (**c**) selectivity of H_2_, (**d**) yield of H_2_, and (**e**) volume fraction of hydrogen generated by porous- and dense-strut SX structures.

During the whole reforming process, the main reactions follow Equations (1) and (2). The total volume, selectivity and conversion rate of the reactants and products were measured and calculated using Equations (3)–(7), as shown in Figure 7. For the dense-strut and hierarchical self-catalytic structure, the structures with the highest surface area, namely the SX-A and SX-A’ structures, exhibit the best reforming efficiency. The conversion rate of CH_4_ decreases with a decrease in the surface area (Figure 7a), indicating a reduction in the reforming efficiency. In addition, temperature is the other decisive factor for the efficiency of the reforming reaction. The reforming efficiency increases with increasing temperature. All values of the indicators, namely α(CH_4_), selectivity (H_2_), selectivity (CO), yield (H_2_) and the volume fraction of H_2_, are the lowest at 500 °C and reach the highest at 900 °C. At low temperatures, such as 500 °C and 600 °C, the reaction hardly occurs due to the insufficient activity of the catalyst. When the temperature increases to 700 °C, the reaction starts and shows a drastic improvement in activity when the temperature reaches 800 °C and 900 °C. This trend indicates that the dense-strut specimen shows the best catalyst efficiency at 900 °C [10]. It is noteworthy that the hierarchical structures, namely SX-A’, SX-B’, SX-C’ and SX-D’, increase the reforming efficiency at lower temperatures. As Figure 7a shows, the conversion rate of CH_4_ increases at all temperatures, and the improvement in the hierarchical structure becomes evident at 800 °C. Due to the improving effect of the hierarchical structure, the conversion rate of CH_4_ becomes comparable at 800 °C and 900 °C for the SX-A’ structure, while the conversion rate of CH_4_ for the SX-A structure at 800 °C is only one-third of that at 900 °C. The improvement in the conversion rate of CH_4_ shows that the hierarchical structure is capable of reducing the temperature for the catalyst effect of Ni to occur.

As for the selectivity of CO and H_2_, the hierarchical structure, especially the SX-A’ specimen, also shows a sharp increase at 800 °C. The selectivity of CO and H_2_ reaches 45% and 47% for the SX-A’ specimen at 800 °C, while the selectivity of CO and H_2_ is less than 5% for the SX-A specimen (Figure 7b,c). As a result, the total volume and yield of H_2_ generated by the hierarchical structure are higher than those generated by the dense-strut specimen (Figure 7d,e). Compared to the dense-strut specimens, the self-catalytic specimens with hierarchical structures (i.e., SX-A’, SX-B’, SX-C’ and SX-D’) show an abrupt increase in the productivity of hydrogen at 700 °C and 800 °C. The total volume fraction of hydrogen produced by SX-A’ increases to 32.95% and 61.76%, respectively, which is much higher than that produced by SX-A (4.21% at 700 °C and 5.16% at 800 °C). However, the total volume of hydrogen becomes similar for dense-strut and hierarchical specimens when the temperature increases to 900 °C.

### 3.4. Microstructural Evolution

**Figure 8:**
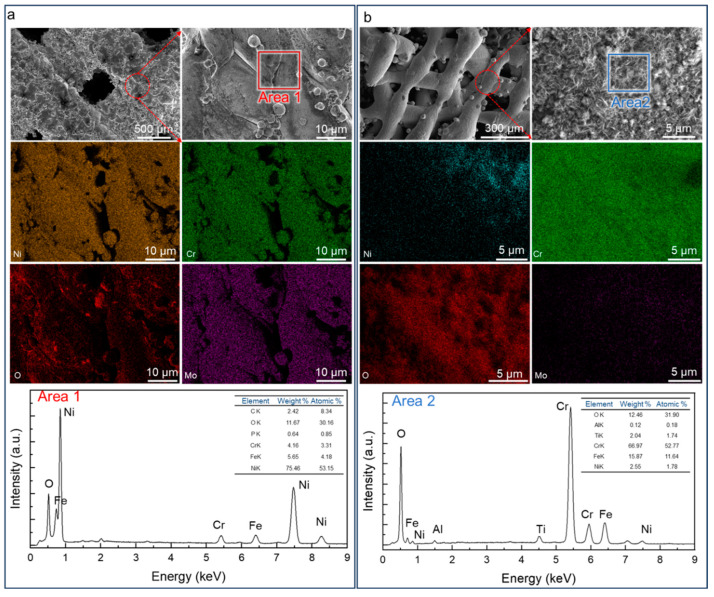
SEM micrographs and EDX elemental analysis result of (**a**) SX-A structure and (**b**) SX-A’ structure after reforming reaction performed subsequently at 500 °C, 600 °C, 700 °C, 800 °C, and 900 °C for 1 h.

The surface morphology of dense-strut and porous-strut SX specimens is further investigated to reveal the microstructural evolution after the reforming reaction, as described in Section 2.5 and shown in Figure 8. Compared to the feedstock powder (Figure 1d), the SX-A specimen shows the concentration of O in the examined area (Figure 8a) after the reforming reaction, and the point analysis result shows a high content of O, indicating the oxidation of the self-catalytic structure during the reforming process. As for the SX-A’ specimen, the SEM characterization reveals the formation of needle-like particles forming on the surface of the porous struts (Figure 8b). The EDX analysis reveals that the needle-like particles contain a high concentration of Cr and O, as the point analysis result shows. Moreover, the other two major elements, Mo and Cr, are detected minorly on the surface of the structure. Compared to the SX-A structure, despite the O element being detected on the surface, the major elements, i.e., Ni, Cr, and Mo, are still the dominant elements. The decrease in Ni and Mo on the surface of the SX-A’ structure is most likely due to the chromium oxide covering the matrix of the struts. The distribution of the elements indicates a layer of the oxide of chromium forming on the surface of the struts in the specimen after the reforming reaction, and more oxidation occurs in the SX-A’ specimen than in the SX-A specimen. The formation of Cr oxide indicates that more reactions between the self-catalytic reactor and the reactants occurred due to the introduction of the hierarchical structure. The hierarchical structure imposes a thorough mixing of the reactants, and the Cr element is preferentially oxidized due to the presence of water vapor.

**Figure 9:**
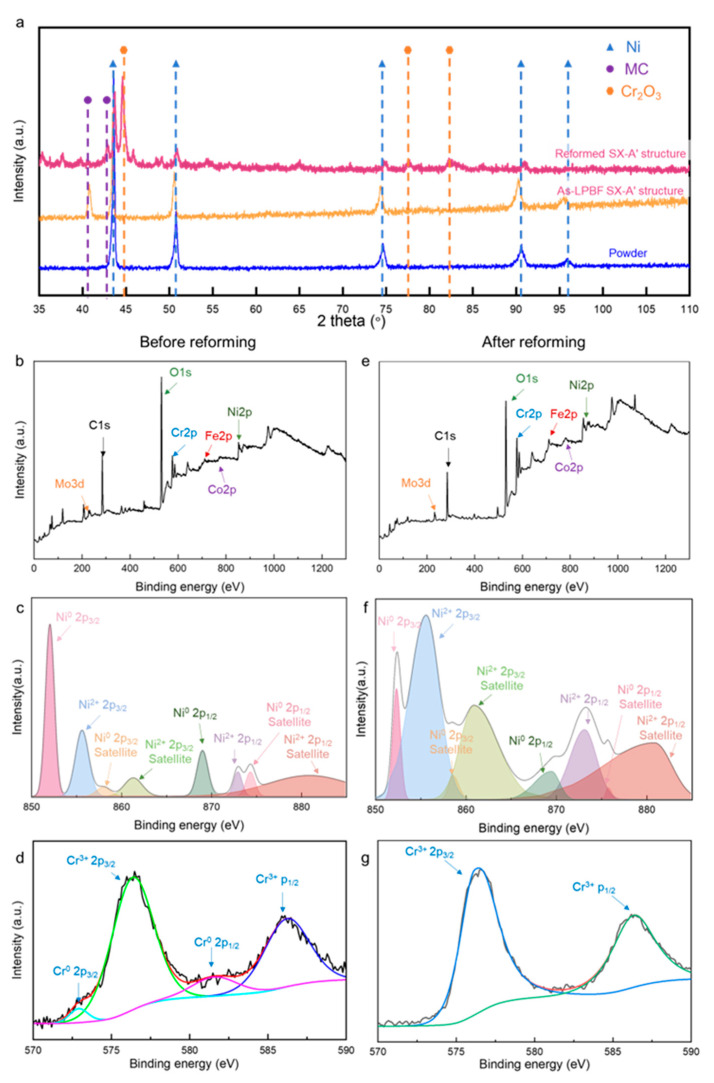
(**a**) XRD pattern of IN625 powder, LPBFed and reformed IN625 self-catalytic structure. XPS spectra of (**b**) all elements, (**c**) Cr elements, (**d**) Ni elements before reforming and (**e**) all elements, (**f**) Cr elements, (**g**) Ni elements after reforming.

To further examine the compositional evolution during the reforming process, XRD is used to examine the feedstock and SX-structured self-catalyst before and after the reforming process. The XRD result confirms that Ni is the only phase detected in the feedstock powder, and a metallic carbide forms in the LPBF-ed SX-A’ specimen, indicating the phase transformation during the LPBF process (Figure 9a). Additionally, the peaks of Cr_2_O_3_, which are not detected in the feedstock powder and bulk specimen, are identified in the XRD spectrum of the reformed SX-A’ structure. As Cr_2_O_3_ is detected in the reformed SX-A’ specimen, this indicates that the oxidation of Cr occurs during the reforming reaction. The XRD result confirms the occurrence of oxidation, which is consistent with the EDX result in Figure 8.

To further examine the surface variation in the SX-A’ specimen during the reforming process, the specimens are tested using XPS. The peaks of the main elements are all detectable before and after the reforming process (Figure 9b,e). From the high-resolution spectrum of Cr 2p, the peak area percentage shows that the SX-A structure has a higher percentage of Cr_2_O_3_ and a minor percentage of Cr in the metallic form [18]. However, a higher percentage of Cr_2_O_3_ in the SX-A’ specimen is detected after the reforming reaction, while the peaks of metallic-form Cr are hardly detected after reforming (Figure 9c,f). The significant decrease in the percentage of metallic-form Cr confirms the oxidation of Cr in the SX-A’ specimen. Additionally, from the Ni 2p spectrum, the peak area percentage shows a high content of metallic-form Ni in the SX-A’ structure before the reforming reaction. In addition, a small quantity of NiO is detected. However, a drastic increase in the content of NiO is detected after the reforming reaction as the peaks corresponding to NiO become larger in the peak area, indicating that Ni also undergoes oxidation during the reforming process [19].

As shown in Figure 7, the hierarchical structure significantly decreases the critical temperature for the catalyst effect of Ni to occur, and the conversion rate of CH_4_ increases significantly for specimens with a hierarchical structure at 800 °C. With the increase in the temperature, the conversion rate increases slightly at 900 °C. For the specimen with a hierarchical structure, higher reaction efficiency induces a higher selectivity and yield of H_2_ compared to the dense-strut specimens (Figure 7b,d). The higher conversion rate of CH_4_ is supposed to induce a higher selectivity of H_2_ and CO. However, the selectivity of CO shows the opposite trend as it is higher in the SX-A structure than in the SX-A’ structure (Figure 7c), indicating that less CO is detected in the product generated by the SX-A’ structure. The decrease in the selectivity of CO is attributable to the side reaction (Equation (2)). The decrease in the selectivity of CO indicates the occurrence of the side reaction in the hierarchical structure. As the hierarchical structure contains a large amount of micro-sized pores, the micro-sized pores lead to the retainment of CO in the hierarchical structure, and the rough surface creates more reaction sites for the side reaction to occur. Therefore, the retained CO reacts more with water vapor and produces more H_2_. In contrast, the dense-strut structure has a smooth surface, meaning that the gaseous product can easily flow through the channel, leaving no time for a side reaction to occur. Therefore, the hierarchical structure also provokes the side reaction and causes more CO to be transformed into H_2_. Therefore, less CO is detected and more H_2_ is generated in the hierarchical structure, leading to less selectivity of CO in SX-A’ than in SX-A specimens. Similarly, it is noteworthy that although the conversion rate of CH_4_ is higher at 900 °C than at 800 °C (Figure 7a), the volume fraction of H_2_ in the product is comparable in the SX-A’ specimens (Figure 7e). The high H_2_ volume fraction is most likely attributable to the extent of the side reaction. As demonstrated in Equation (2), the side reaction is exothermic, and therefore, the CO-to-H_2_ side reaction occurs more favorably at 800 °C than at 900 °C, leading to more CO being transformed into H_2_. As a result, the volume fraction of H_2_ for the SX-A’ specimen is comparable at 800 °C and 900 °C.

Additionally, the XRD and XPS results (Figure 9) reveal that the Ni-based self-catalytic reactor oxidizes during the reforming process. The XRD patterns reveal the formation of Cr_2_O_3_ after the reforming process (Figure 9a). As shown in Figure 9a, the peaks of Ni (PDF#45-1027) are all detected in the feedstock powder, LPBF specimen and reformed specimens. However, the peak of Cr_2_O_3_ (PDF#38-1479) was only detected in the reformed specimen, indicating the oxidation of Cr elements during the reforming process. From the XPS spectra, the peaks of the major elements of IN625 were detected before and after the reforming process (Figure 9b,e). As a distinct feature of transition elements, the satellite peaks of Ni 2p spectra are detected, and only the photoelectron peaks are analyzed. The peak area percentage of Ni^0^ 2p_3/2_ decreased significantly from 28.4% to 5.1%, while that of Ni^2+^ 2p_3/2_ increased significantly from 16.3% to 32.8% before and after the reforming process (Figure 9c,f). The increase in the peak area percentage of Ni^2+^ 2p_3/2_ indicates the oxidation of the Ni elements. The oxidation of Ni is mainly concentrated on the surface of the struts, and thus, Ni oxides are not detected by XRD. Similarly, the oxidation of Cr is also confirmed by the increasing peak area percentage of Cr^3+^ 2p_3/2_ and the peak of Cr^0^ 2p_3/2_ is hardly distinguishable (Figure 9d,g). The oxidation of the Ni-based catalyst inevitably decreases the activity [20] due to the deactivation of the Ni elements and the formation of the oxide layer. The formation of Cr_2_O_3_ reduces the reaction sites. With the progress of the oxidation, needle-like Cr_2_O_3_, as shown in Figure 8, develops into a compact layer [21], separating the CH_4_ and Ni catalysts, leading to the failure of the catalyst system. Therefore, as the hierarchical structure provokes the reforming reaction at a lower temperature, the oxidation of the self-catalytic system is prolonged without sacrificing the productivity of H_2_, highlighting another advantage of the hierarchical structure.

**Figure 10:**
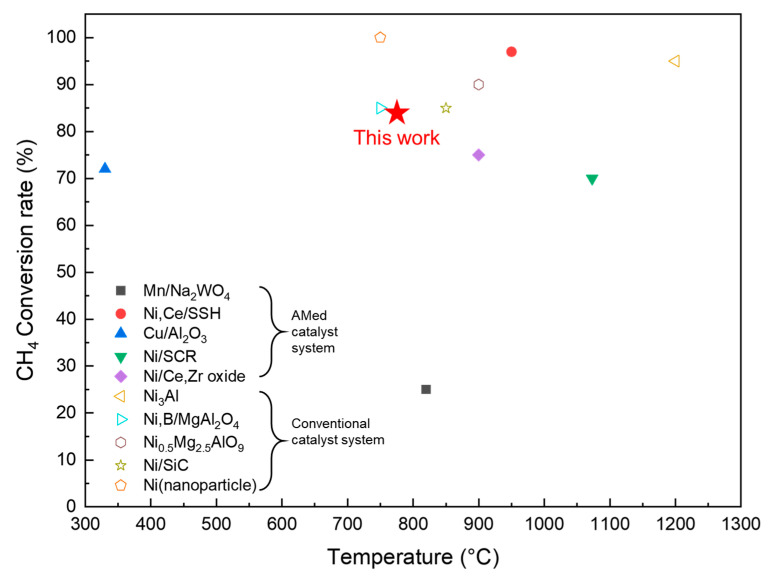
The catalytic performance of the conventional and AMed Ni-based catalytic systems [22,23,24,25,26,27,28,29,30,31,32].

In this study, the AMed Ni-based self-catalytic reactor exhibits an excellent methane conversion rate (>80%) at 800 °C. Figure 10 shows the catalytic performance, indicated by the methane conversion rate, of the conventional and AMed Ni-based catalytic systems. Compared to the conventional Ni-based catalyst system, self-catalytic reactors with a hierarchical structure show comparable properties, except for the systems that use nano-sized particles as catalysts. Compared to other AMed Ni-based catalytic systems, the self-catalytic reactor decreases the optimum temperature of the reforming process. It is noteworthy that some conventional catalytic systems use modified catalysts (such as nano-sized Ni particles or Ni_3_Al) and exhibit a nearly 100% conversion rate. Modifying the materials of a self-catalytic reactor is likely to further improve the catalytic properties.

## 4. Conclusions

In this study, a hierarchical structure is incorporated into different mixer structures (SX structures) to increase the efficiency of the methane reforming process. The formability, microstructure and catalyst performance of the hierarchical structure are investigated. The main conclusions are summarized as follows:

Decreasing the energy input leads to the balling of a single track and introduces lack-of-fusion defects in the bulk specimens. The lack-of-fusion defects lead to the formation of micro-sized pores on the strut of the hierarchical self-catalytic reactor.

The self-catalytic reactor with a hierarchical structure is much lighter than that without a hierarchical structure, with a decrease in the mass by an average of nearly 50%. Also, the introduction of the hierarchical structure significantly increases the surface area and porosity of the self-catalytic reactor, although the strength of the structure decreases.

The SX structure with the highest surface area and porosity, namely the SX-A structure, shows the best reforming efficiency. The introduction of the hierarchical structure increases the reforming efficiency at all temperatures. For the self-catalytic reactor without a hierarchical structure, the highest reforming efficiency occurs at 900 °C, while those with a hierarchical structure exhibit a comparable reforming efficiency at 800 °C. This improvement could prolong the lifespan of the self-catalytic reactor.

The introduction of the hierarchical structure shows a significant improvement in the reforming reaction. The improvement of the hierarchical structure is mainly attributable to (1) the increase in the reaction site due to a larger surface area, (2) more mixing of the reactants for the main reaction to occur and (3) the retainment of the gases for the side reaction to proceed.

The findings of this study proves that a high-specific-surface-area structure fabricated by AM can effectively improves the catalytic performance of a self-catalytic reactor, revealing the potential of the AMed self-catalytic reactor in industrial applications. However, the oxidation and long-term stability of the AMed self-catalytic reactor still needs further optimization, which requires the innovation of its materials and structure.

## Figures and Tables

**Table 1:** Processing parameters for the fabrication of the specimens.

	Continuous/Dense Parts	Discontinuous/Porous Parts
Single-track deposition	/	Power: 80–220 W Scanning speed: 700–1300 mm/s
Bulk sample deposition	/	Power: 120 W Scanning speed: 800 mm/s Hatch distance: 0.21 mm Layer thickness: 40 μm
SX-structured deposition	Power: 280 W Scanning speed: 950 mm/s Hatch distance: 0.11 mm Layer thickness: 40 μm	Power: 120 W Scanning speed: 800 mm/s Hatch distance: 0.21 mm Layer thickness: 40 μm

## Data Availability

The original contributions presented in the study are included in the article/Supplementary Materials, further inquiries can be directed to the corresponding author.

## Supplementary Materials

The following supporting information can be downloaded at: https://www.mdpi.com/article/10.3390/ma18061350/s1, File S1: The selection of flow rate of the reactants. References [10,33] are cited in the Supplementary Materials.
