# Supplementary material for: Improving Steam Methane Reforming Efficiency via Hierarchical Structure in Additively Manufactured Ni-Based Self-Catalytic Reactors
Source: Materials (Basel). 2025 Mar 19;18(6):1350. doi: 10.3390/ma18061350 (PMC11943580; doi:10.3390/ma18061350)
Supplement: Supplementary file 1 [file materials-18-01350-s001.zip › materials-3493657-supplementary.pdf]

The selection of the flow rate of the reactants is derived from two classic references [1] and [2].

In ref [1], the optimum flow rate of CH_4 is 40 ml/min for an 8mm diameter catalyst, and therefore, the flow rate is converted to 50 ml/L for the 10 mm diameter catalyst in this study. In ref [2], the critical H_2O/CH_4 ratio for the reforming process is no less than 2:1.

Based on the aforementioned information, the molar flow rate of CH_4 ($n(CH_4)$) is calculated as

$n(CH_4)=50mL/min \div 22400mL/mol \approx 0.00223mol/min$ (the volume of 1mol of gas under standard conditions is 22.4L):

$n(H_2O)=2\times n(CH4)=2\times 0.00223=0.00446mol/min$,

and therefore, the mass of the deionized water

$m(H_2O)=n(H_2O)\times 18g/mol=0.00446\times 18\approx 0.0803$ g/min. And the flow rate of water $Q(H_2O)= =0.0803g/min \div 1g/mL \approx 0.0803mL/min$. Therefore, the flow rate of water is determined to be 0.081 ml/min.

The flow rate of N_2 (10 mL/min) is the recommended value of the gas analyzer.

[1] Q. Wei, H. Li, G. Liu, Y. He, Y. Wang, Y.E. Tan, D. Wang, X. Peng, G. Yang, N. Tsubaki, Metal 3D printing technology for functional integration of catalytic system, Nat Commun 11 (2020) 4098. https://doi.org/10.1038/s41467-020-17941-8.

[2] J. Rostrup-Nielsen, Steam reforming of hydrocarbons. A historical perspective, in: X. Bao, Y. Xu (Eds.), Stud Surf Sci Catal, Elsevier, 2004: pp. 121–126. https://doi.org/https://doi.org/10.1016/S0167-2991(04)80038-7.
